# Mental Distress Among Females Following 2021 Abortion Restrictions in Texas

**DOI:** 10.1001/jamanetworkopen.2025.9576

**Published:** 2025-05-12

**Authors:** Jusung Lee, Kari White, Vanessa K. Dalton

**Affiliations:** 1 Department of Public Health, College for Health, Community and Policy, University of Texas at San Antonio; 2 Resound Research for Reproductive Health, Austin, Texas; 3 Department of Obstetrics and Gynecology, University of Michigan, Ann Arbor; 4 Program on Women’s Healthcare Effectiveness Research, University of Michigan, Ann Arbor

## Abstract

**Importance:**

Understanding whether abortion restrictions are associated with poor mental health is crucial for mitigating policy, public health, or clinical interventions.

**Objective:**

To quantify changes in frequent mental distress among females in Texas, following implementation of the 2021 Texas Heartbeat Act Senate Bill 8 (SB8), which banned abortions upon detection of embryonic cardiac activity.

**Design, Setting, Participants:**

This cross-sectional study was conducted using 2012 to 2022 data from the Behavioral Risk Factor Surveillance System (BRFSS), a population-based representative survey. Participants were aged 18 to 44 years and included females in Texas, males in Texas, and females in other states. Data were analyzed from May 2024 to February 2025.

**Exposure:**

Implementation of SB8 in Texas in September 2021.

**Main Outcomes and Measures:**

The primary outcome was frequent mental distress, defined as 14 days or more of poor mental health during the past 30 days. Difference-in-differences models were used to examine the association between SB8 implementation and changes in frequent mental distress among females aged 18 to 44 years in Texas compared with males in Texas and females from other states.

**Results:**

This study included 79 609 individuals (age proportion, 18 to 29 years [43.9%], 30 to 39 years [38.3%], 40 to 44 years [17.8%]; 15 614 females in Texas [25.5%]; 14 500 males in Texas [26.1%]; 49 495 females in other states [48.4%]). Between 2012 and 2022, frequent mental distress increased from 14.2% (95% CI, 13.2%-15.2%) to 21.9% (95% CI, 19.4%-24.4%) in 15 614 females in Texas and increased from 11.1% (95% CI, 10.2%-12.0%) to 15.0% (95% CI, 13.1%-16.9%) in 14 500 males in Texas. SB8 implementation in 2021 was associated with an adjusted increase of 6.8 (95% CI, 3.0-10.6) percentage points among females compared with males. In a stratified analysis by age group, SB8 was associated with larger increases in frequent mental distress among females aged 18 to 29 years (9.8 [95% CI, 3.1-16.7] percentage points) and females aged 30 to 39 years (7.4 [95% CI, 2.0-12.9] percentage points) compared with males. In adjusted models comparing females in Texas with females in other states, SB8 was associated with an increase of 5.3 (95% CI, 1.7-9.0) percentage points compared with females in 5 pooled states (Arkansas, Indiana, Kentucky, Mississippi, Oklahoma) and an increase of 6.1 (95% CI, 2.0-10.2) percentage points compared with females in California in frequent mental distress.

**Conclusions and Relevance:**

In this repeated cross-sectional study, there was a significant association between SB8 implementation and an increase in frequent mental distress. Disproportionately higher increases among younger people could be associated with higher levels of anticipated or actual need for abortion care or less ability to overcome barriers to care, including travel. These findings signal that state abortion policy may negatively affect mental health.

## Key Points

**Question:**

Is there an association between abortion restrictions and mental distress in Texas?

**Findings:**

In this cross-sectional study of 79 609 individuals, the implementation of severe abortion restrictions in 2021 was associated with an increase 6.8 percentage points frequent mental distress among females in Texas compared with males and an increase of 5.3 percentage points among females in Texas compared with females in states that had not yet passed severe restrictions.

**Meaning:**

These findings suggest that Texas’s abortion restrictions were associated with increases in mental distress among females of reproductive age, especially among younger individuals who may have less ability to overcome barriers to abortion care.

## Teaser

This cross-sectional study examines the changes in mental health among females in Texas after implementation of the Texas Heartbeat Act in September 2021 and compares it with the mental health of males in Texas and females in other states.

## Introduction

In September 2021, Texas implemented the Texas Heartbeat Act Senate Bill 8 (SB8), which essentially banned abortion after detection of embryonic cardiac activity, making Texas the most restrictive state in the US.^1^ Because embryonic cardiac activity can occur as early as 5 to 6 weeks from the start of the last menstrual cycle, this law was followed by 57% reduction in facility-based abortions occurring in Texas.^2^ Subsequent to the US Supreme Court decision in *Dobbs v Jackson Women’s Health Organization* in June 2022 and as of February 2025, 16 states have implemented outright or near complete abortion bans.^3^ Because Texas banned abortion earlier than other states, the Texas experience provides much of what is known about the consequences of abortion bans on health. Following implementation of SB8, out-of-state travel for abortion among Texans increased.^4^ However, there is growing evidence that not everyone is able to obtain abortion care, even in medically complicated pregnancies, sometimes resulting in adverse health outcomes.^5,6,7^

Concerns that abortion bans could negatively impact mental health have been raised. Prior research using data from multiple states has shown that a range of abortion restrictions, including the *Dobbs v Jackson Women’s Health Organization *decision, are associated with poorer mental health.^8,9,10,11^ One recent study reported that the *Dobbs v Jackson Women’s Health Organization *decision was associated with a 10% increase in the prevalence of mental distress among females living in states where abortion was likely to be banned compared with females living in states where abortion was protected.^11^ One limitation of pooled, multistate data is that it is very difficult to fully account for state-specific confounders that may track abortion restrictions, such as poverty policy or access to mental health services. Accordingly, we used a difference-in-differences analysis to examine changes in self-reported mental distress following Texas SB8 among females compared with males in Texas. To account for the COVID-19 pandemic or other contemporaneous events, we performed additional comparisons using females in other states as controls.

## Methods

This population-based, repeated cross-sectional study was conducted using the Behavioral Risk Factor Surveillance System (BRFSS) (2012, 2014, 2016, and 2018 to 2022). BRFSS is a state-centric and population-representative sample of noninstitutionalized adults aged 18 years and older. The survey instruments measured health and demographic information, including race and ethnicity, relying on participants’ self-reports. Individuals aged 18 to 44 years who reported their mental health experiences in the past 30 days were included in the study population. The Strengthening the Reporting of Observational Studies in Epidemiology (STROBE) reporting guideline were followed in this study. The study was reviewed and deemed not regulated by the University of Texas at San Antonio institutional review board because BRFSS was deidentified, secondary public-use data.

The outcome of interest was frequent mental distress, defined as participants reporting 14 or more days of poor mental health (including stress, depression, and problems with emotions) during the previous 30 days. The measure was created using responses to the following question: “Now thinking about your mental health, which includes stress, depression, and problems with emotions, for how many days during the past 30 days was your mental health not good.”^12,13,14^

We used a 2-way fixed-effects difference-in-differences design to evaluate changes in frequent mental distress among females in Texas compared with males in Texas after SB8 implementation in 2021. Female was defined as respondents who indicated female sex at birth, and male was defined as respondents who indicated male sex at birth. Although self-identified gender has been collected since 2016, there is a high degree of missingness. As a sensitivity analysis, we conducted models accounting for self-reported gender identity (eTable 1 in Supplement 1) and found similar outcomes.

Texas SB8 was passed on May 19, 2021, and went into effect on September 1 of the same year. For our main model, we defined the preexposure period as January 2012 to August 2021 (period 1) and the postexposure period as September 2021 to December 2022 (period 2). We also conducted a sensitivity analysis using May 2021 (date of SB8 passage) as the start of the exposure period (eTable 2 in Supplement 1). To account for COVID-19 or other unidentified events, we conducted additional models using 2 alternative comparison groups: (1) 5 states with trigger laws (state laws that automatically banned abortion when *Roe v Wade* was overturned) and similar COVID-19 policies (Arkansas, Indiana, Kentucky, Mississippi, and Oklahoma) and (2) California because it is similar to Texas with respect to the racial and ethnic composition of the population.^4,15,16,17,18^ A difference-in-differences regression using a linear probability model was performed to estimate changes in frequent mental distress in Texas compared with controls, with and without accounting for study covariates. Study covariates included age, race and ethnicity, education, employment, marital status, check-up within 1 year, body mass index, health status, and a year-fixed effect (eMethods in Supplement 1).^9,19,20,21^ Complex survey weights and design variables were accounted in all analyses.

### Statistical Analysis

After visually assessing trends in frequent mental distress from 2012 to 2022, we tested the validity of the parallel trends assumption in our preexposure period (period 1) by interacting the policy indicator with the year variable. We then evaluated the association between SB8 implementation and frequent mental distress using a difference-in-differences approach comparing our treatment group (females) with the control group (males). We modeled unadjusted and adjusted changes in frequent mental distress among females in Texas and males in Texas before and after SB8 implementation. Next, we performed subgroup analyses by stratifying the sample by age groups. To account for major events that occurred during the study period, such as the COVID-19 pandemic and the *Dobbs v Jackson Women’s Health Organization* decision, we tested the validity of the parallel trends assumption (eFigures 1 and 2 in Supplement 1) and examined changes in frequent mental distress among females in Texas with females from the 2 other comparison groups. Statistical significance was set at *P* < .05 and Stata/SE version 18.0 (StataCorp) and R version 4.1.1 (R Project for Statistical Computing) were used for formal statistical analysis and data visualization, respectively. Data were analyzed from May 2024 to February 2025.

## Results

This study included 79 609 individuals (age proportion, 18 to 29 years [43.9%], 30 to 39 years [38.3%], 40 to 44 years [17.8%]; 15 614 females in Texas [25.5%]; 14 500 males in Texas [26.1%]; 49 495 females in other states [48.4%]). Among a total of 15 614 female participants in Texas, the mean annual prevalence of frequent mental distress was 14.2% (95% CI, 13.2%-16.9%) at baseline and 21.9% (95% CI, 19.4%-24.4%) after SB8, indicating an increase of 7.7 percentage points (Table 1). Among the 14 500 male participants, there was an increase of 3.9 percentage points between period 1 (11.1% [95% CI, 10.2%-12.0%]) and period 2 (15.0% [95% CI, 13.1%-16.9%]). Participant characteristics by sex at birth in Texas are shown in eTable 3 in Supplement 1.

**Table 1.** Difference-in-Differences Estimates in Frequent Mental Distress Among Females Compared With Males Before and After SB8 in Texas for the Total Sample and by Age Group^a^

Sample	Mental distress, % (95% CI)				Unadjusted % change (95% CI), percentage points	*P* value	Adjusted % change (95% CI), percentage points	*P* value
	Males		Females					
	Period 1^b^	Period 2^b^	Period 1^b^	Period 2^b^				
Total sample	11.1 (10.2 to 12.0)	15.0 (13.1 to 16.9)	14.2 (13.2 to 15.2)	21.9 (19.4 to 24.4)	3.8 (0.4 to 7.2)	.03	6.8 (3.0 to 10.6)	<.001
Age group, y								
18-29	11.3 (10.0 to 12.7)	17.8 (14.5 to 21.2)	16.0 (14.3 to 17.7)	27.6 (23.0 to 32.2)	5.1 (−0.9 to 11.1)	.10	9.8 (3.1 to 16.7)	.005
30-39	10.8 (9.3 to 12.3)	12.3 (9.7 to 14.8)	12.7 (11.4 to 14.0)	19.4 (15.9 to 22.8)	5.2 (−0.2 to 8.7)	.03	7.4 (2.0 to 12.9)	.008
40-44	11.0 (8.9 to 13.2)	13.3 (9.4 to 17.3)	13.1 (11.0 to 15.2)	15.0 (10.7 to 19.3)	−0.5 (−7.0 to 6.1)	.89	0.3 (−6.2 to 6.9)	.93

^a^ The adjusted model accounted for study covariates, such as age, race and ethnicity, education, employment, marital status, check-up within 1 year, body mass index, health status, and a year fixed effect. Subgroup analyses were performed by stratifying the sample by age groups. All models accounted for the complex survey weights and design variables.

^b^ Period 1 indicates January 2012 to August 2021, and period 2 indicates September 2021 to December 2022.

**Figure 1.**
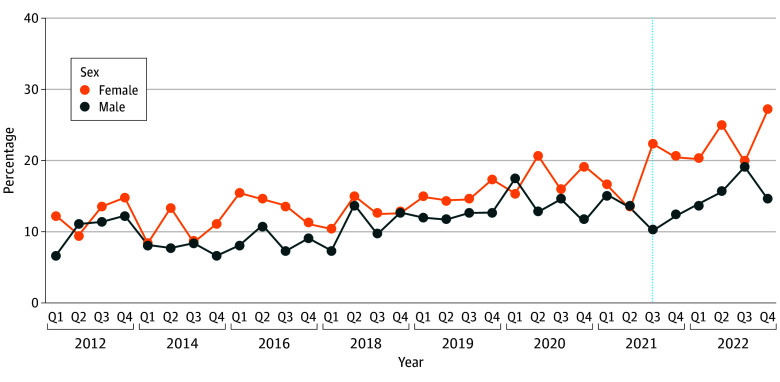
Unadjusted Frequent Mental Distress in Texas 2012 to 2022 A vertical line represents the third quarter (Q) of 2021 when Texas Senate Bill 8 (September 2021) went into effect.

**Figure 2.**
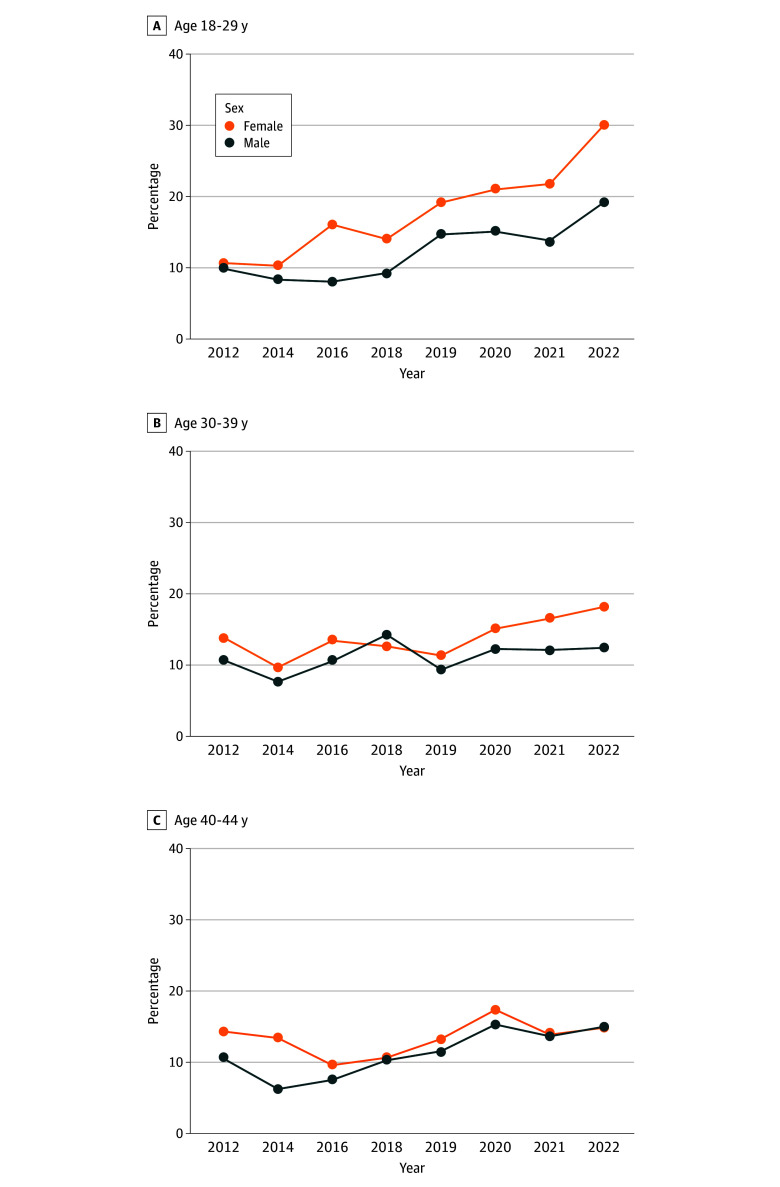
Unadjusted Frequent Mental Distress in Texas 2012 to 2022 by Age Group

Baseline trends in mental distress among females in Texas and males in Texas during the preexposure period did not differ significantly, fulfilling the parallel trends assumption (eFigure 2 in Supplement 1). However, following SB8, there were disproportionately higher increases in frequent mental distress among females compared with males (Figure 1). The adjusted change from the difference-in-differences analysis found that SB8 was associated with an increase of 6.8 (95% CI, 1.6 to 8.7) percentage points among females compared with males (Table 1). Stratified models revealed differences in the change by age group (Figure 2). For instance, SB8 was associated with an increase of 9.8 (95% CI, 0.6 to 13.6) percentage points among females compared with males aged 18 to 29 years but no significant difference between females and males aged 40 to 44 years (0.3 [95% CI, −6.2% to 6.9%] percentage points) (Table 1).

Models comparing reproductive age female respondents in Texas with female respondents in other states found similar increases in mental distress associated with the implementation of SB8. When comparing Texas respondents to those in the pooled sample of 5 states, SB8 implementation was associated with an increase of 5.3 (95% CI, 1.7-9.0) percentage points in frequent mental distress (Table 2). Similarly, compared with females in California, SB8 was associated with an increase of 6.1 (95% CI, 2.0-10.2) percentage points in frequent mental distress. In the subgroup analysis stratified by age, SB8 was associated with an increase of 7.3 (95% CI, 0.5-14.0) percentage points compared with females in pooled states and an increase of 13.8 (95% CI, 6.6-21.1) percentage points compared with females in California (pooled states and California, respectively) in frequent mental distress among young females aged 18 to 29 years (Table 3). Participant characteristics in these supplemental models are shown in eFigure 3 and eTable 4 in Supplement 1.

**Table 2.** Difference-in-Differences Estimates in Frequent Mental Distress Among Females in Texas Compared With Females From Other Control States Before and After SB8

Sample	Mental distress, % (95% CI)				Unadjusted % change (95% CI), percentage points	*P* value	Adjusted % change (95% CI), percentage points	*P* value
	5 states (Arkansas, Indiana, Kentucky, Mississippi, Oklahoma)		Texas					
	Period 1^b^	Period 2^b^	Period 1^b^	Period 2^b^				
Total sample	21.2 (20.5 to 21.8)	26.2 (24.7 to 27.8)	14.2 (13.2-15.2)	21.9 (19.4 to 24.4)	2.7 (−0.5 to 5.8)	.10	5.3 (1.7 to 9.0)	.004
Age group, y								
18-29	22.3 (21.3 to 23.4)	31.0 (28.4 to 33.7)	16.0 (14.3 to 17.7)	27.6 (23.0 to 32.2)	2.9 (−2.7 to 8.6)	.31	7.3 (0.5 to 14.0)	.03
30-39	20.1 (19.2 to 21.1)	23.7 (21.5 to 26.0)	12.7 (11.4 to 14.0)	19.4 (15.9 to 22.8)	3.1 (−1.3 to 7.6)	.17	5.0 (−0.3 to 10.3)	.06
40-44	20.4 (19.1 to 21.7)	20.5 (17.7 to 23.2)	13.1 (11.0 to 15.2)	15.0 (10.7 to 19.3)	1.7 (−3.9 to 7.4)	.55	2.8 (−3.2 to 8.8)	.36

^a^ The estimates represent changes in Texas compared with females in other states. Subgroup analyses were performed by stratifying the sample by age groups. The adjusted model accounted for study covariates, such as age, race and ethnicity, education, employment, marital status, check-up within 1 year, body mass index, health status, and a year fixed effect. All models accounted for complex survey weights and design variables.

^b^ Period 1 indicates January 2012 to August 2021, and period 2 indicates September 2021 to December 2022.

**Table 3.** Difference-in-Differences Estimates in Frequent Mental Distress Among Females in Texas Compared With Females From California Before and After SB8^a^

Sample	Mental distress, % (95% CI)				Unadjusted % change (95% CI), percentage points	*P* value	Adjusted % change (95% CI), percentage points	*P* value
	California		Texas					
	Period 1^b^	Period 2^b^	Period 1^b^	Period 2^b^				
Total sample	15.2 (14.3 to 16.0)	19.0 (16.9 to 21.2)	14.2(13.2 to 15.2)	21.9 (19.4 to 24.4)	3.9 (0.3 to 7.4)	.03	6.1 (2.0 to 10.2)	.003
Age group, y								
18-29	17.8 (16.4 to 19.3)	20.4 (17.0 to 23.8)	16.0 (14.3 to 17.7)	27.6 (23.0 to 32.2)	9.0 (2.9 to 15.1)	.004	13.8 (6.6 to 21.1)	<.001
30-39	13.6 (12.3 to 14.9)	16.9 (13.7 to 20.1)	12.7 (11.4 to 14.0)	19.4 (15.9 to 22.8)	3.4 (−1.7 to 8.4)	.19	4.6 (−1.4 to 10.5)	.13
40-44	12.3 (10.6 to 14.1)	20.3 (14.8 to 25.9)	13.1 (11.0 to 15.2)	15.0 (10.7 to 19.3)	−6.2 (−13.7 to −1.4)	.11	−6.1 (−13.9 to 1.6)	.12

^a^ The estimates represent changes in Texas compared with females in other states. Subgroup analyses were performed by stratifying the sample by age groups. The adjusted model accounted for study covariates, such as age, race and ethnicity, education, employment, marital status, check-up within 1 year, body mass index, health status, and a year fixed effect. All models accounted for complex survey weights and design variables.

^b^ Period 1 indicates January 2012 to August 2021, and period 2 indicates September 2021 to December 2022.

## Discussion

The findings of this study suggest that the implementation of Texas SB8 was associated with an increase of 6.8 percentage points in frequent mental distress among females in Texas compared with males in Texas. Furthermore, the observed increase in frequent mental distress was most notable among younger females. These patterns persisted when comparing females of reproductive age in Texas with females in states where abortion bans were anticipated but had not yet been enacted. The consistency of these findings suggests that the restricted access to abortion was associated with poorer mental health.

Upon implementation of SB8 in 2021, Texas became the first state to severely restrict access to abortion. As such, many early studies about the consequences of abortion restrictions report on the experience in Texas.^2,6,22,23,24,25,26,27^ Studies have reported that SB8 was associated with an unexpected increase in infant and neonatal deaths and higher rates of maternal morbidity following required changes in treatment of obstetrical complications.^28^ Studies in Texas and elsewhere have reported an increase in worry or distress about the ability to get needed pregnancy care, legal ramifications of seeking or obtaining abortion care, and logistical difficulties getting out-of-state abortion care.^2,8,11,22,25,26^ Our study adds to a growing body of evidence that has found abortion restrictions to be associated with poorer mental health.

The finding that increases in frequent mental distress were more pronounced among younger females was not surprising. Even before early abortion bans were enacted, young people reported experiencing numerous challenges in accessing abortion care, including difficulty traveling for abortion care and a lack of support from adults.^29^ Younger people also experience higher rates of unwanted pregnancies and more often use abortion care compared with older age groups.^30,31^ The increasingly restrictive policy environment creates even larger obstacles for young people seeking abortion care, a group less able to overcome the barriers than their older counterparts.^32^ Consequently, young people may experience distress about realized or anticipated barriers to abortion care to a greater degree than other age groups.

### Strengths and Limitations

This study has numerous strengths including its large sample size, use of rigorous quasi-experimental methods, and use of different control populations. However, this study has several limitations. It is not possible to completely exclude that another unidentified event or policy, such as COVID-19, is the true cause of the observations. However, the fact that we found similar estimates using 3 different control populations lessens this concern considerably. Next, we acknowledge that our study relied on a single self-reported measure of distress, and we do not have information about the duration of symptoms. Finally, given our study’s observational design, this study does not prove that SB8 causes mental distress. Future studies examining abortion restrictions and mental health in other states could strengthen the evidence of a causal relationship.

## Conclusions

This cross-sectional study found that the implementation of SB8, a Texas law that banned abortion at 6 weeks’ gestation, was significantly associated with an increase in frequent mental distress among reproductive aged females in Texas, especially females aged 18 to 29 years. These findings suggest that restricting access to abortion may be associated with poorer mental health, particularly among young people.

## Notes

Supplement 1. **eTable 1.** Difference-in-Differences Estimates in Frequent Mental Distress Among Females Compared With Males Before and After SB8 in Texas, 2016 to 2022 With Adjustment of Gender Identity **eTable 2.** Sensitivity Analysis for Difference-in-Differences Estimates in Frequent Mental Health in Females in Texas Compared With Males in Texas and Females From Control States, Before and After SB8 Legislation in Texas (Period 2 Set From May 2021 When SB8 Was Enacted)
eMethods.
**eTable 3.** Participant Characteristics in Texas **eTable 4.** Participant Characteristics of Females From Other Control States **eFigure 1.** Unadjusted Frequent Mental Distress in Control States 2012 to 2022 **eFigure 2.** Parallel Trends Assumption Test From January 2012 to August 2021 **eFigure 3.** Unadjusted Frequent Mental Distress Among Females in Texas Compared With Females in Control States 2012 to 2022 by Age Group

Supplement 2.
Data Sharing Statements
