# Supplementary material for: Mental Distress Among Females Following 2021 Abortion Restrictions in Texas
Source: JAMA Netw Open. 2025 May 12;8(5):e259576. doi: 10.1001/jamanetworkopen.2025.9576 (PMC12070235; doi:10.1001/jamanetworkopen.2025.9576)
Supplement: Supplement 1. — eTable 1. Difference-in-Differences Estimates in Frequent Mental Distress Among Females Compared With Males Before and After SB8 in Texas, 2016 to 2022 With Adjustment of Gender Identity eTable 2. Sensitivity Analysis for Difference-in-Differences Estimates in Frequent Mental Health in Females in Texas Compared With Males in Texas and Females From Control States, Before and After SB8 Legislation in Texas (Period 2 Set From May 2021 When SB8 Was Enacted) eMethods. eTable 3. Participant Characteristics in Texas eTable 4. Participant Characteristics of Females From Other Control States eFigure 1. Unadjusted Frequent Mental Distress in Control States 2012 to 2022 eFigure 2. Parallel Trends Assumption Test From January 2012 to August 2021 eFigure 3. Unadjusted Frequent Mental Distress Among Females in Texas Compared With Females in Control States 2012 to 2022 by Age Group [file jamanetwopen-e259576-s001.pdf]

Supplemental Online Content

Lee J, White K, Daltom V. Mental distress among females following 2021 abortion restriction in Texas. *JAMA Netw Open*. 2025;8(5):e259576. doi:10.1001/jamanetworkopen.2025.9576

eTable 1. Difference-in-Differences Estimates in Frequent Mental Distress Among Females Compared With Males Before and After SB8 in Texas, 2016 to 2022 With Adjustment of Gender Identity

eTable 2. Sensitivity Analysis for Difference-in-Differences Estimates in Frequent Mental Health in Females in Texas Compared With Males in Texas and Females From Control States, Before and After SB8 Legislation in Texas (PERIOD 2 Set From May 2021 When SB8 Was Enacted)

eMethods.

eTable 3. Participant Characteristics in Texas

eTable 4. Participant Characteristics of Females From Other Control States

eFigure 1. Unadjusted Frequent Mental Distress in Control States 2012 to 2022

eFigure 2. Parallel Trends Assumption Test From January 2012 to August 2021

eFigure 3. Unadjusted Frequent Mental Distress Among Females in Texas Compared With Females in Control States 2012 to 2022 by Age Group

This supplemental material has been provided by the authors to give readers additional information about their work.

© 2025 Lee J et al. *JAMA Network Open*.

eTable 1. Difference-in-Differences Estimates in Frequent Mental Distress Among Females Compared With Males Before and After SB8 in Texas, 2016 to 2022 With Adjustment of Gender Identity

	Males % (95% CI)		Females % (95% CI)		Unadjusted change		Adjusted change	
	Period 1 (Jan 2012 – Aug 2021)	Period 2 (Sept 2021 – Dec 2022)	Period 1 (Jan 2012 – Aug 2021)	Period 2 (Sept 2021 – Dec 2022)	% (95% CI)	P-value	% (95% CI)	P-value
Frequent mental distress	12.0 (10.8, 13.1)	15.3 (13.1, 16.9)	15.0 (14.0, 16.5)	21.9 (19.4, 24.4)	3.6 (0.0, 7.2)	0.048	3.6 (0.0, 7.1)	0.044

Note: Gender identity was reported as transgender (male to female, female to male, gender nonconforming) and no transgender. The missing values (>20%) were imputed using multiple imputations with chained equations. The adjusted model accounted for study covariates, such as age, gender identity, race and ethnicity, education, employment, marital status, check-up within 1 year, body mass index, and a year fixed effect. Complex survey weights and design variables were accounted.

© 2025 Lee J et al. *JAMA Network Open*.

eTable 2. Sensitivity Analysis for Difference-in-Differences Estimates in Frequent Mental Health in Females in Texas Compared With Males in Texas and Females From Control States, Before and After SB8 Legislation in Texas (PERIOD 2 Set From May 2021 When SB8 Was Enacted)

	Males in Texas		Five states (Arkansas, Indiana, Kentucky, Mississippi, Oklahoma)		California	
	% (95% CI)	P-value	% (95% CI)	P-value	% (95% CI)	P-value
Frequent mental health	6.3 (2.7, 9.8)	<.001	3.9 (0.5, 7.2)	0.021	4.9 (1.2, 8.6)	0.010

Note: Analyses using linear probability models were adjusted for study covariates, such as age, race and ethnicity, education, employment, marital status, check-up within 1 year, body mass index, health status, and a year fixed effect. Complex survey weights and design variables were accounted.

© 2025 Lee J et al. *JAMA Network Open*.

eMethods. Difference-in-Differences Model Specification

Difference-in-Differences model specification for males and females in Texas

1) $Y_{it} = \beta_0 + \beta_1 TexasSB8_t + \beta_2 Female_i + \beta_3(TexasSB8_t * Female_i) + \gamma X_{it} + T_t + \epsilon_{it}$

Where Y_{it} represents frequent mental distress for individual i in calendar year t from 2012 to 2022. $TexasSB8_t$ is an indicator for one (after Texas SB8 went into effect in September 2021) and zero otherwise. $Female_i$ is an indicator equal to one for an individual identified as female at birth and zero for an individual identified as male at birth. Females are the target population of Texas SB8 to restrict their access to abortion or reproductive care. X_{it} is a vector of demographics and control variables (age, race and ethnicity, marital status, education, employment, and check-up within 1 year.). $T_t$is a year fixed effect, and $\epsilon_{it}$is a random error.

Difference-in-Differences model specification for females in Texas compared to females in control states

2) $Y_{ist} = \beta_0 + \beta_1 TexasSB8_t + \beta_2 Texas_s + \beta_3(TexasSB8_t * Texas_s) + \gamma X_{ist} + State_s + T_t + \epsilon_{ist}$

Where Y_{ist} represents frequent mental distress for individual i from a state s in calendar year t from 2012 to 2022. $State_s$ is a state fixed effect.

© 2025 Lee J et al. *JAMA Network Open*.

eTable 3. Participant Characteristics in Texas

			Period 1 (Jan 2012 – Aug 2021)			Period 2 (Sept 2021 – Dec 2022)		
		Total	Females	Males	P-value	Females	Males	P-value
		wt % (n)	wt % (n)	wt % (n)		wt % (n)	wt % (n)	
Overall		100 (30,114)	49.5 (12,638)	50.5 (11,285)		49.1 (2,976)	50.9 (3,215)	
Age, years					0.030			0.072
	18–29	44.6 (11,394)	43.6	46.1		41.0	45.6	
	30–39	37.9 (12,422)	38.1	36.6		40.3	36.0	
	40–44	17.5 (6,298)	17.3	17.3		18.6	18.3	
Race/ethnicity					0.002			0.28
	Non–Hispanic Whites	36.0 (13,546)	34.9	38.1		32.4	34.9	
	Non–Hispanic Blacks	11.3 (2,604)	12.5	10.3		12.3	9.9	
	Non–Hispanic Others	9.1 (2,441)	8.7	8.6		11.2	10.6	
	Hispanics	43.6 (11,477)	43.9	43.0		44.2	44.5	
Marital Status					<.001			0.010
	Currently married	42.6 (13,607)	44.6	40.7		44.9	40.5	
	Divorced/widowed/ separated	9.8 (3,493)	11.5	8.8		9.3	7.2	
	Unmarried couple	7.8 (2,257)	8.4	7.0		8.6	8.5	
	Never married	39.8 (10,459)	35.5	43.5		37.2	43.7	
Education					<.001			0.002
	Less than high school	15.2 (3,204)	16.3	15.3		13.2	11.9	
	High school	28.5 (7,868)	25.5	31.0		25.5	33.1	
	Some college	31.6 (8,100)	33.1	30.2		33.3	30.0	
	College degree or above	24.7 (10,810)	25.1	23.5		28.0	25.0	
Employment					<.001			<.001
	No	32.1 (9,101)	44.4	20.8		38.6	23.1	
	Yes	67.8 (20,713)	55.6	79.2		58.4	76.9	
Check-up within 1 year					<.001			<.001
	No	39.5 (11,651)	35.2	43.9		33.0	44.5	

© 2025 Lee J et al. *JAMA Network Open*.

	Yes	60.5 (17,877)	64.8	56.1		67.0	55.5	
Body Mass Index					<.001			<.001
	Underweight	2.5 (543)	1.5	2.7		2.6	2.0	
	Normal weight	36.4 (8,836)	30.0	39.3		29.8	36.5	
	Overweight	32.2 (8,645)	38.5	26.9		37.2	27.0	
	Obese	28.8 (8,536)	30.0	31.1		30.4	34.5	
Health status					0.015			0.51
	Not good	12.3 (3,899)	11.2	12.9		13.1	14.0	
	Good	87.7 (26,083)	88.8	87.1		86.9	56.0	
Year								
	2012	11.7 (2,978)						
	2014	11.9 (4,447)						
	2016	12.2 (2,952)						
	2018	12.4 (3,469)						
	2019	12.8 (3,711)						
	2020	13.1 (4,022)						
	2021	12.2 (3,575)						
	2022	13.7 (4,960)						

© 2025 Lee J et al. *JAMA Network Open*.

eTable 4. Participant Characteristics of Females From Other Control States

		Five states (Arkansas, Indiana, Kentucky, Mississippi, Oklahoma)			California		
		Total	Period 1 (Jan 2012 – Aug 2021)	Period 2 (Sept 2021 – Dec 2022)	Total	Period 1 (Jan 2012 – Aug 2021)	Period 2 (Sept 2021 – Dec 2022)
		wt % (n)	wt % (n)	wt % (n)	wt % (n)	wt % (n)	wt % (n)
Overall		(34,559)	(28,859)	(5,700)	(14,936)	(12,890)	(2,046)
Age, years							
	18–29	43.9 (11,972)	44.2	42.7	42.9 (5,739)	43.0	42.4
	30–39	37.8 (14,487)	37.6	38.7	39.1 (6,235)	39.1	39.3
	40–44	18.3 (8,100)	18.2	18.7	18.0 (2,962)	17.9	18.3
Race/ethnicity							
	Non–Hispanic Whites	67.3 (24,388)	**67.9**	**64.5**	28.1 (5,199)	**28.3**	**26.7**
	Non–Hispanic Blacks	16.2 (5,152)	**16.3**	**15.7**	5.0 (792)	**5.3**	**3.6**
	Non–Hispanic Others	7.2 (2,414)	**6.7**	**9.5**	22.0 (2,473)	**21.7**	**23.5**
	Hispanics	9.3 (2,577)	**9.0**	**10.3**	44.9 (6,427)	**44.7**	**46.2**
Marital Status							
	Currently married	44.9 (17,278)	**45.0**	**44.3**	41.0 (6,345)	**41.2**	**40.2**
	Divorced/widowed/ separated	13.2 (5,140)	**13.6**	**11.8**	8.9 (1,525)	**9.1**	**8.1**
	Unmarried couple	6.2 (1,852)	**6.0**	**6.9**	9.9 (1,478)	**10.0**	**8.9**
	Never married	35.7 (10,153)	**35.4**	**37.1**	40.2 (5,505)	**39.7**	**42.7**
Education							
	Less than high school	11.6 (2,425)	**12.3**	**8.7**	16.2 (1,953)	**16.4**	15.4
	High school	28.5 (8,846)	**28.0**	**30.4**	22.5 (2,826)	**22.4**	22.9
	Some college	36.9 (11,378)	**36.8**	**37.3**	32.7 (3,721)	**33.1**	30.5
	College degree or above	23.0 (11,844)	**22.9**	**23.6**	28.6 (6,307)	**28.2**	31.2
Employment							
	No	39.2 (12,340)	**40.4**	**34.1**	45.0 (6,100)	45.9	39.5
	Yes	60.8 (21,966)	**59.6**	**65.9**	55.0 (8,740)	54.1	60.5
Check-up within 1 year							

© 2025 Lee J et al. *JAMA Network Open*.

	No	30.7 (10,143)	**31.9**	**25.6**	35.0 (5,277)	35.9	30.2
	Yes	69.3 (23,595)	**68.1**	**74.4**	65.0 (9,558)	64.1	69.8
Body Mass Index							
	Underweight	2.6 (680)	**2.7**	**2.2**	3.8 (440)	**4.0**	**2.7**
	Normal weight	34.7 (10,526)	**35.4**	**31.3**	44.4 (5,812)	**44.9**	**41.7**
	Overweight	25.9 (8,119)	**25.8**	**26.4**	27.2 (3,510)	**29.9**	**28.8**
	Obese	36.8 (11,480)	**36.2**	**40.1**	24.6 (3,086)	**24.2**	**26.8**
Health status							
	Not good	14.7 (4,820)	**14.7**	14.7	12.7 (1,854)	12.4	14.0
	Good	85.3 (29,683)	**85.2**	85.3	87.3 (13,078)	87.6	86.0
Year							
	2012	12.3 (5,547)			12.6 (2,666)		
	2014	12.3 (4,667)			11.5 (1,492)		
	2016	12.7 (4,449)			14.9 (2,634)		
	2018	11.8 (3,712)			11.8 (2,099)		
	2019	12.8 (4,227)			13.9 (2,266)		
	2020	12.2 (3,932)			11.4 (896)		
	2021	12.5 (3,803)			13.9 (1,317)		
	2022	13.4 (4,333)			10.0 (1,566)		

Note: Numbers in bold indicate statistical significance at p<.05 for comparing sample characteristics with those of Texas for each period.

© 2025 Lee J et al. *JAMA Network Open*.

eFigure 1. Unadjusted Frequent Mental Distress in Control States 2012 to 2022

1.1. Five states (AR, IN, KY, MS, OK) vs TX

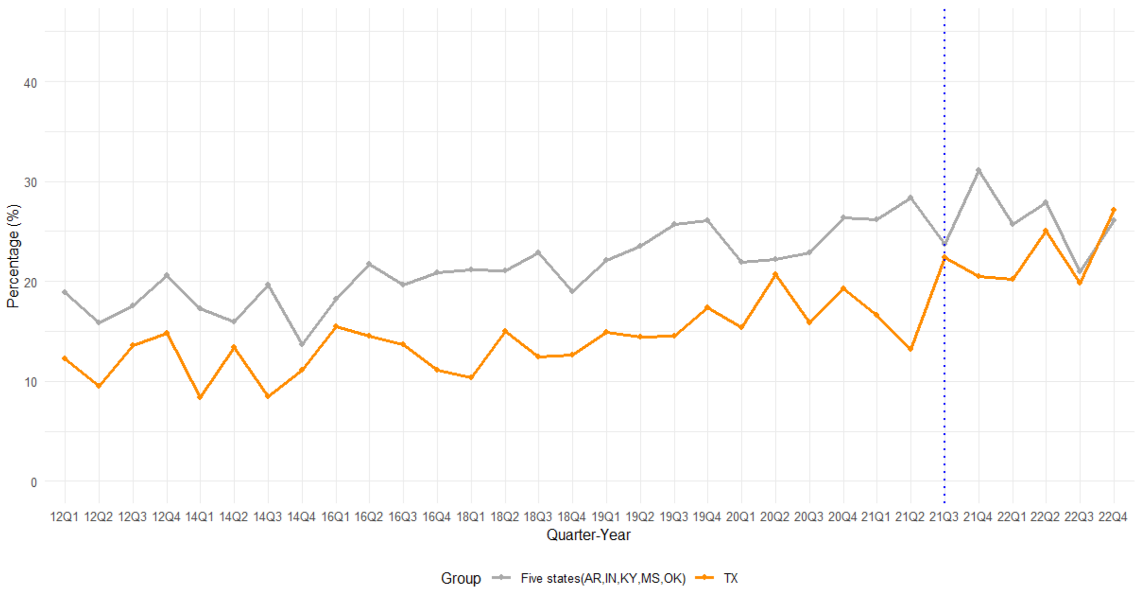

1.2. CA vs TX

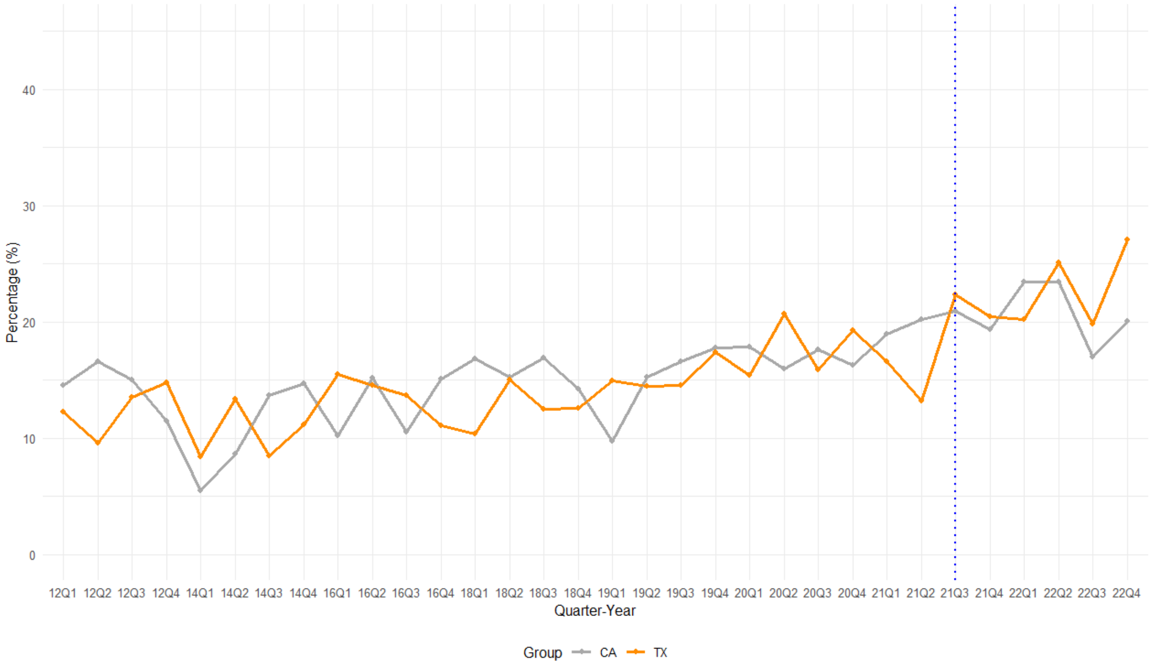

© 2025 Lee J et al. *JAMA Network Open*.

eFigure 2. Parallel Trends Assumption Test From January 2012 to August 2021

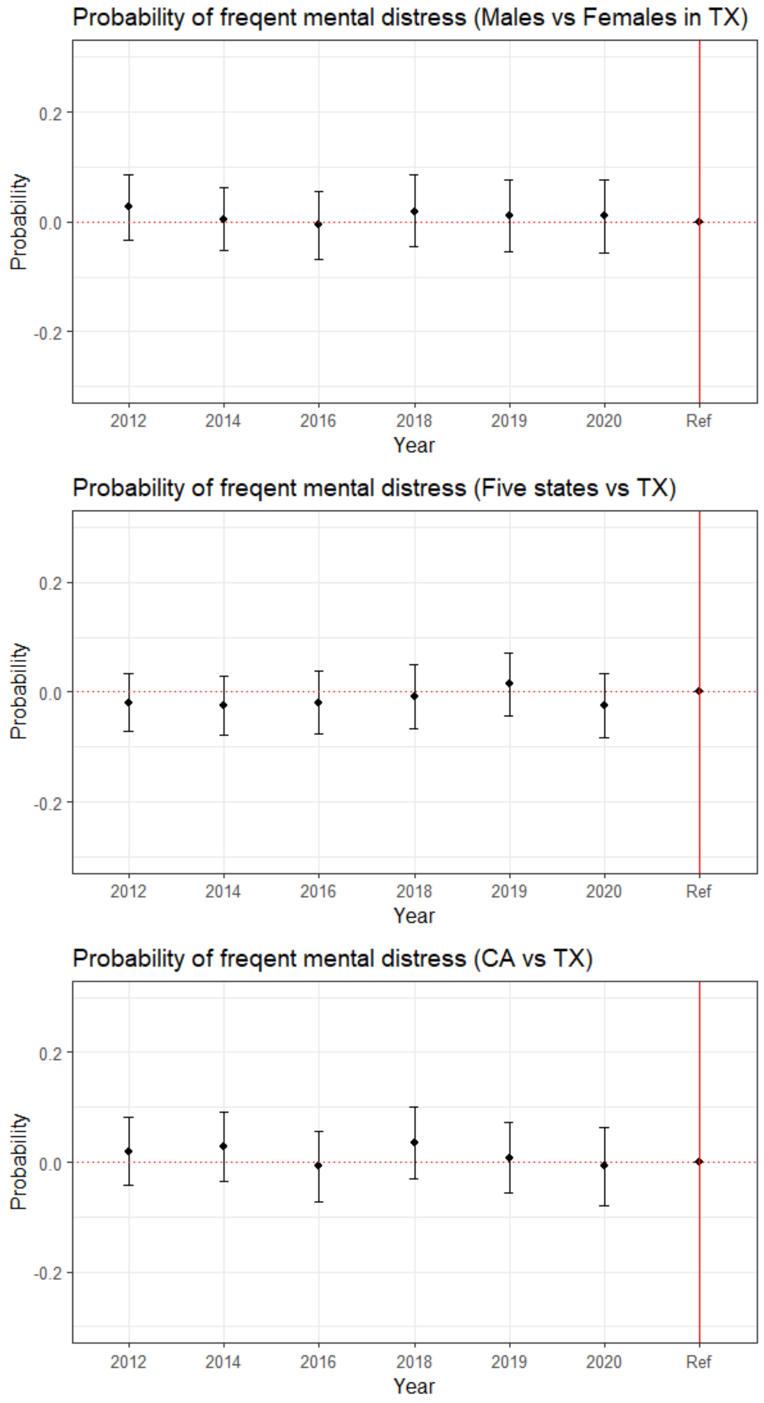

Note: Coefficients in the figure are adjusted difference (95% CI) between females in Texas compared to males in Texas and females from control states, holding the difference at the reference (1/2021-8/2021) at zero. All estimated differences between groups were not statistically different from 0, suggesting that a parallel trend assumption has been met during the baseline.

© 2025 Lee J et al. *JAMA Network Open*.

eFigure 3. Unadjusted Frequent Mental Distress Among Females in Texas Compared With Females in Control States 2012 to 2022 by Age Group

3.1. Five states (AR, IN, KY, MS, OK) vs TX

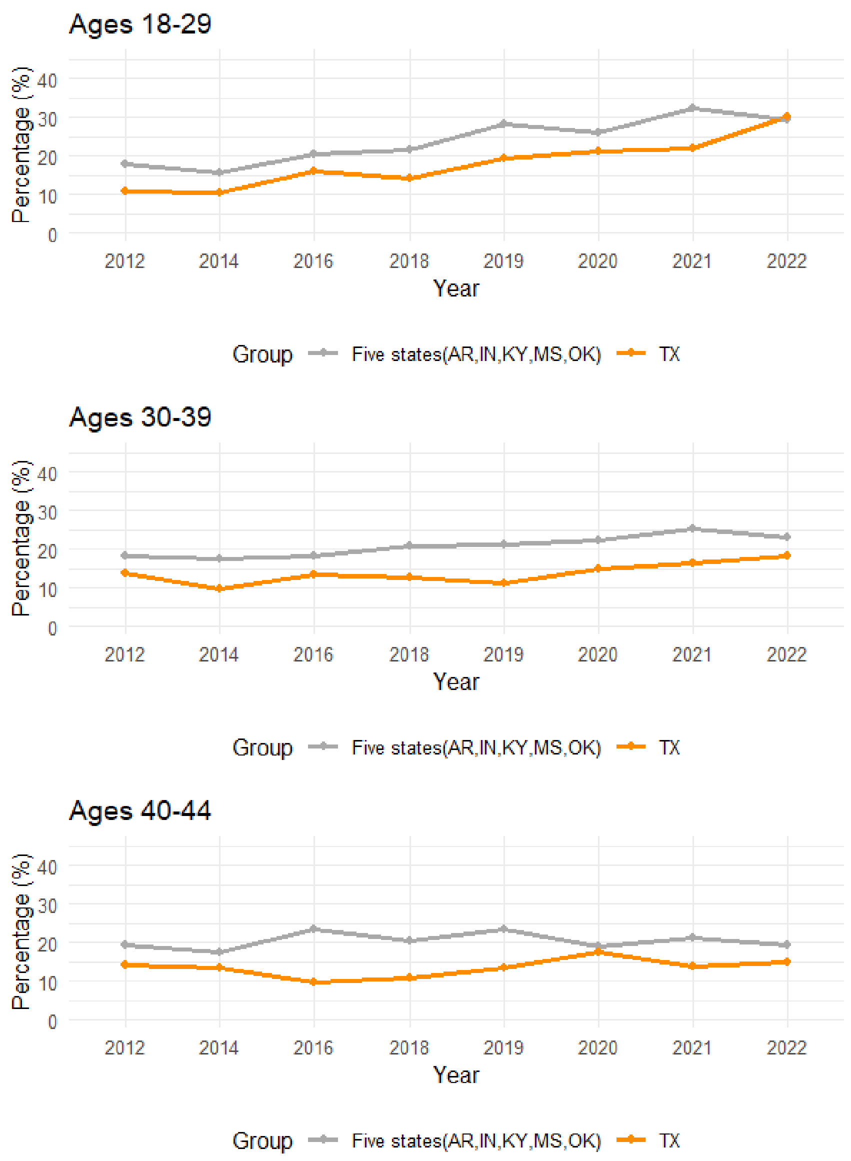

3.2. CA vs TX

© 2025 Lee J et al. *JAMA Network Open*.

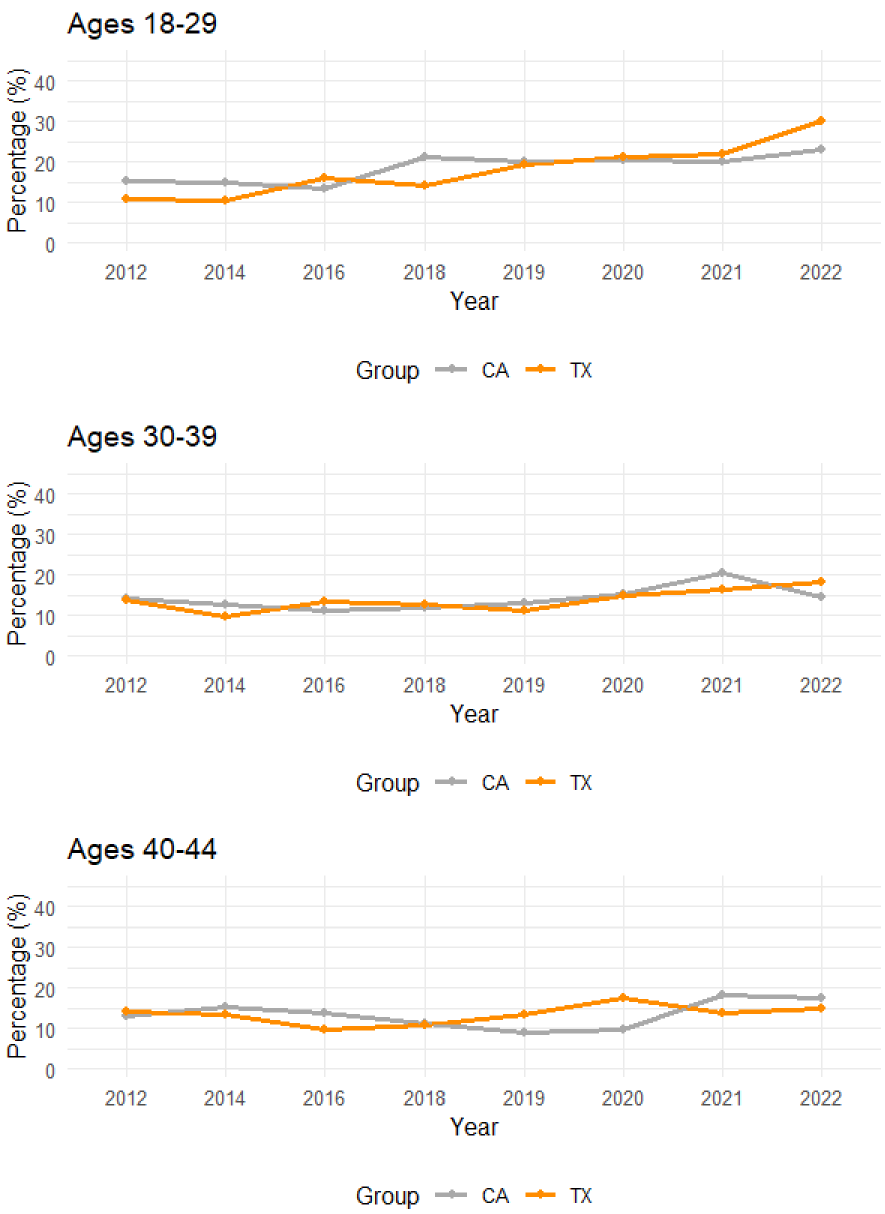

© 2025 Lee J et al. *JAMA Network Open*.
