# Supplementary material for: Mental Distress Among Females Following 2021 Abortion Restrictions in Texas
Source: JAMA Netw Open. 2025 May 12;8(5):e259576. doi: 10.1001/jamanetworkopen.2025.9576 (PMC12070235; doi:10.1001/jamanetworkopen.2025.9576)
Supplement: Supplement 2. — Data Sharing Statements [file jamanetwopen-e259576-s002.pdf]

Data Sharing Statement

Lee. Mental Distress Among Females Following 2021 Abortion Restrictions in Texas. *JAMA Netw Open*. Published May 12, 2025. doi:10.1001/jamanetworkopen.2025.9576

Data
Data available: Yes
Data types: Deidentified participant data
How to access data: https://www.cdc.gov/brfss/annual_data/annual_data.htm
When available: beginning date: 10-02-2024

Supporting Documents
Document types: Other (please specify)
Additional Information: The code used in this study may be available from the corresponding author upon reasonable request.
How to access documents: The code used in this study may be available from the corresponding author upon reasonable request (jusung.lee@utsa.edu)
When available: With publication

Additional Information
Who can access the data: Anyone can access the data through the Behavioral Risk Factor Surveillance System of the Centers for Disease Control and Prevention.
Types of analyses: The data is publicly available.
Mechanisms of data availability: It is readily available to the public.
